# Osseous Tumor of the Hand: Finger Enchondroma

**Published:** 2015-01-13

**Authors:** Tom Reisler, Stephen L. Viviano, Mark Granick

**Affiliations:** Department of Surgery, Division of Plastic Surgery, Rutgers New Jersey Medical School, Newark, NJ

**Keywords:** hand bone tumor, enchondroma, digit, demineralized bone matrix, Maffucci syndrome

## DESCRIPTION

A 17-year-old child with a known left ring finger proximal phalanx enchondroma comes to the office a year after sustaining a pathological fracture, which was treated with splinting. He says he has been in pain since the incident occurred and is here today for a definitive diagnosis and treatment.

## QUESTIONS

1. **How common are enchondromas?**
2. **What are the characteristic features seen on hand x-ray in this disease?**
3. **How do enchondromas usually present?**
4. **How are enchondromas managed?**

## DISCUSSION

Enchondromas are benign tumors of the bone that are thought to develop from fragments of cartilage near the central physis. Microscopic examination shows benign clusters of hyaline cartilage surrounded by lamellar bone with varying calcification. They occur equally in men and women. Solitary enchondromas typically develop during the second or third decade of life. They account for 12% to 14% of benign bone neoplasms and 3% to 10% of osseous neoplasms in general. In addition, enchondromas are the most common primary bone tumor in the hand, accounting for as many as 90% of bone tumors seen in the hand. The tumor has a predilection for the small bones of the hands and feet. Approximately 35% of all enchondromas arise in the hand. Of these, approximately 50% are in the proximal phalanx, followed in frequency by the metacarpal and middle phalanx.^1^^-^4

Multiple enchondromas may present in 3 disorders: Ollier disease, Maffucci syndrome, and metachondromatosis. Both Ollier disease and Maffucci syndrome are nonhereditary sporadic disorders. Features that distinguish Maffucci syndrome from Ollier disease are the presence of hemangiomas and lymphangiomas; and the association with a higher risk of CNS, pancreatic, and ovarian malignancies. Metachondromatosis is an autosomal-dominant inherited disease associated with both multiple osteochondromas and enchondromas.^5^

**Figure 1:**
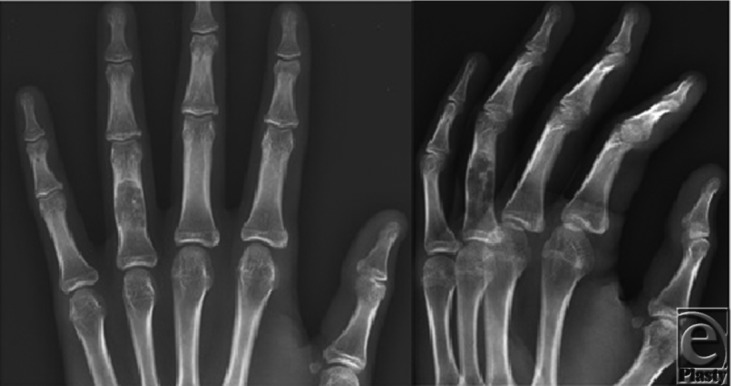
Radiographs of the left hand with an expansile lytic lesion within the midshaft of the ring finger proximal phalanx with endosteal scalloping and thinning of the surrounding cortex.

Radiographically, enchondromas appear as well-defined areas of central lucency in the metaphyseal or diaphyseal portion of the bone. They may expand enough to cause thinning and endosteal scalloping of the cortex. Stippling and punctate calcifications may also be seen within the areas of lucency (Fig 1).^2^

Enchondromas are often asymptomatic and discovered incidentally on imaging studies. However, pathologic fractures occurring in the area of the tumor may lead to the diagnosis.^3^

**Figure 2:**
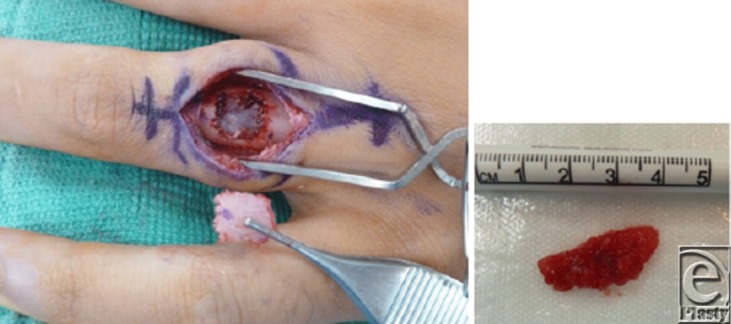
Clinical photograph demonstrating a longitudinal dorsal incision over the proximal phalanx to expose the enchondroma. Cortical bone flap elevated, exposing the underlying enchondroma (*left*). Two centimeters of gelatinous mass of enchondroma curetted and sent to frozen section analysis (*right*). The enchondroma cavity was filled in with a bone graft of demineralized bone matrix and the bone flap was placed back into the window.

**Figure 3:**
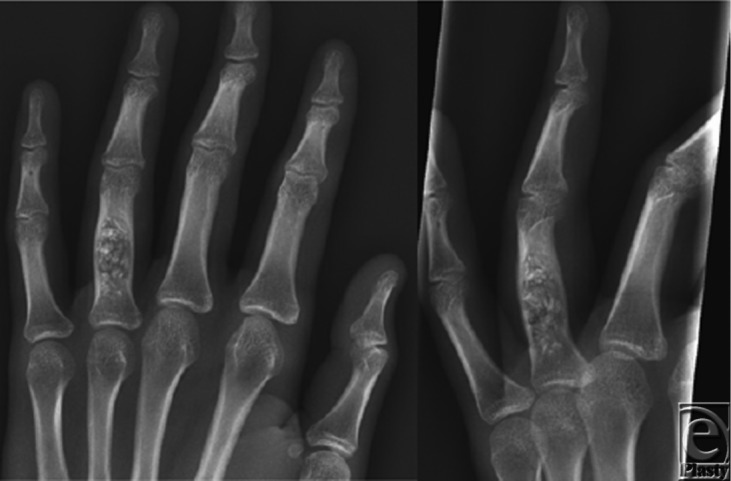
Radiographs of the left hand. Two months' interval from the time of curettage and bone grafting of ring finger midshaft proximal phalanx. There is no pathological fracture or new lytic lesion.

Management of patients with a pathologic fracture usually begins with a period of immobilization to allow for fracture healing. This may then be followed by surgery, which most commonly involves open biopsy, curettage of the entire lesion, bone grafting or bone substitution, and fixation (Fig 2). Recurrence following curettage is infrequent but occurs in 2% to 15% of patients. Because of this, patients should have periodic radiographic screening after surgery at intervals of 6 months, 1 year, and 2 years after initial surgery.^2^ Small enchondromas found incidentally in x-ray study can be followed conservatively with serial x-rays (Fig 3).^6^
